# Intravitreal Conbercept with Grid/Focal Photocoagulation for the Treatment of Diabetic Macular Edema: A Systematic Review and Meta-Analysis

**DOI:** 10.1155/2022/2256779

**Published:** 2022-02-24

**Authors:** Xiaoyan Sun, Wei Wei

**Affiliations:** ^1^Department of Ophthalmology, Nanjing Hospital of Chinese Medicine Affiliated to Nanjing University of Chinese Medicine, Nanjing, 210022 Jiangsu, China; ^2^First Clinical Medical College, Nanjing University of Chinese Medicine, Nanjing, 210046 Jiangsu, China; ^3^Department of Ophthalmology, Jiangsu Province Hospital of Chinese Medicine, Nanjing, 210004 Jiangsu, China

## Abstract

Diabetic macular edema (DME) is the main cause of blindness in individuals with diabetes mellitus (DM). This meta-analysis compared the effectiveness and safety of macular grid/focal photocoagulation with and without conbercept in the treatment of DME. Studies were identified through systematic searches of PubMed, Embase, Cochrane Library, China National Knowledge Infrastructure database, Wanfang Data Knowledge Service Platform, and VIP Information Resource Integration Service Platform from their earliest records to June 2021. Twelve articles involving 2600 patients with DME were included. Results showed that patients receiving conbercept with macular grid/focal photocoagulation had a statistically significant reduction in central macular thickness (CMT) over macular grid/focal photocoagulation alone at 1 month and 3 months post procedure. Compared with the control group, the combination therapy group had a significantly increased level of effectiveness and best-corrected visual acuity (BCVA) compared with the control group. The combination therapy group significantly increased the level of effectiveness and best-corrected visual acuity (BCVA) compared with the control group. Conbercept with macular grid/focal photocoagulation was more effective than macular grid/focal photocoagulation alone in terms of functional outcomes for DME treatment.

## 1. Introduction

Diabetic retinopathy (DR) is acknowledged to be one of the serious microvascular complications of diabetes mellitus (DM) and a leading cause of vision loss in the working-age population in most countries [1]. It brings about disastrous personal and socioeconomic consequences, despite being potentially preventable and treatable [2]. Diabetic macular edema (DME) is caused by disruption of the blood-retinal barrier leading to retinal thickening around the fovea, due to long-term hyperglycemia. The overall prevalence of DME in the PREVAIL study was 5.4%, and the overall prevalence of visual impairment was 1.9% [3]. Therapeutic options for treating DME include intravitreal injection of anti-vascular endothelial growth factor (VEGF), intravitreal steroid, laser photocoagulation, and vitrectomy. Clinicians need to consider certain therapeutic interactions when deciding on the treatment to use. Alessandro et al.[4] showed that in the treatment of naïve DME patients, intravitreal dexamethasone implant demonstrated a better functional response in patients with the presence of serous retinal detachment and ellipsoid zone integrity and absence of vitreomacular alterations. For some diabetic patients in specific patient groups, including pregnant women (in consideration of risk to the fetus and pregnancy), perioperative cataract patients, and persons with recent cardiovascular events, intravitreal steroids may be first choice therapy [5]. Since 1980s, focal/grid laser photocoagulation has been the standard treatment for DME, reducing the risk of blindness and increasing the possibility of vision gain as compared to no treatment [6]. Focal/grid laser photocoagulation is effective in stabilizing and protecting the remaining vision. However, its ability to reverse vision loss is poor. Macular laser photocoagulation prevents microaneurysms from leaking into the macular and improves oxygenation. Photocoagulation and intravitreal therapy have been shown to reduce the number of injections required [7]. Grid and/or focal laser photocoagulation is the first-line therapy for patients with non-central-involved DME (NCI-DME). Anti-VEGF therapy is preferred for patients with centrally involved DME (CI-DME) and moderate visual impairment [7, 8] Many scholars believe that intravitreal injections have evolved to be the better therapy of choice for treating DME as an alternative to macular laser photocoagulation [7]. However, researchers at DRCR.net [9] pointed out that among eyes with good visual acuity (VA) and CI-DME, there was no significant difference in vision loss at two years regardless of the eyes were initially managed with aflibercept or with laser photocoagulation or observation and given aflibercept only on the worsening of the VA. Observation without treatment, unless VA worsens, may be a reasonable strategy for CI-DME. VEGF is a vascular permeability factor that can induce neovascularization and destroy tight capillary wall connections. It is considered as the main factor involved in proliferative diabetic retinopathy (PDR) neovascularization [10]. Conbercept was approved by the China Food and Drug Administration in 2013 and entered basic medical insurance in 2017. It is a recombinant fusion protein with high affinity to both VEGF-A and VEGF-B isoforms and placental growth factor independently developed in China. It has been widely used as a first-line drug for the treatment of DME for many years in China. Many studies have concluded that intravitreal conbercept (IVC) is effective and safe for the treatment of DME. However, there is no systematic evaluation of the therapeutic effect and safety of IVC with macular focal/grid laser photocoagulation versus macular focal/grid laser photocoagulation alone in DME. Therefore, a systematic review and meta-analysis were carried out to appraise the efficacy and safety of conbercept and macular focal/grid laser photocoagulation in the treatment of DME.

## 2. Materials and Methods

This research was registered at PROSPERO (https://www.crd.york.ac.uk/prospero/, registration number CRD42021266444). This systematic review and meta-analysis were conducted according to the PRISMA guidelines (http://prisma-statement.org/).

### 2.1. Database and Search Strategies

We searched PubMed, Embase, Cochrane Library, China National Knowledge Infrastructure (CNKI) database, Wanfang Data Knowledge Service Platform, and VIP Information Resource Integration Service Platform (cqvip) for randomized controlled trials (RCTs) with human participants, published from their earliest records to June 31, 2021. A search strategy that combined Medical Subject Headings and entry terms was adopted to capture as many studies as possible. The search terms were as follows: “Diabetic retinopathy;” “Diabetic Retinopathies;” “Retinopathies, Diabetic;” “Retinopathy, Diabetic;” “Diabetic macular edema;” “Macular Edema;” “Edema, Macular;” “Cystoid Macular Edema, Postoperative;” “Macular Edema, Cystoid;” “Edema, Cystoid Macular;” “Central Retinal Edema, Cystoid;” “Cystoid Macular Edema;” “conbercept;” and “photocoagulation.” The studies were limited to Chinese and English languages. The inclusion criteria were as follows: (1) randomized controlled trials (RCTs); (2) the type of disease was DME; (3) the treatments were conbercept with focal/grid retinal photocoagulation and focal/grid retinal photocoagulation alone; and (4) therapeutic efficacy indicators such as best-corrected visual acuity (BCVA), central macular thickness (CMT), effectiveness rate, and complications. The exclusion criteria were as follows: (1) duplicated articles; (2) studies without available data; (3) animal studies; (4) summary of the meeting, comments, letters, etc.; and (5) meta-analysis and systematic evaluation.

### 2.2. Data Extraction and Quality Evaluation

Two researchers (S and W) independently reviewed the titles and abstracts of all the retrieved articles identified through literature search and assessed the studies. Any disagreements were adjudicated through discussion to validate the accuracy. They independently extracted data from the included articles as follows: (1) first author's name, (2) year of publication, (3) study design, (4) treatment regimens, (5) baseline information, and (6) the number of events in each study. The modified Jadad scale was used to evaluate the quality of the included articles. The scoring system of the modified Jadad scale gave 2 points for properly assigned, 1 point for unclearly assigned, and 0 for inappropriately assigned randomization method and blinding method. For the withdrawal and exit method, 1 point was given if it was clearly described and 0 point if it was not clearly described in the manuscript. The scores ranged from one to seven, with 1–3 for low-quality studies and 4–7 for high-quality studies [11].

### 2.3. Risk of Bias Assessment

Two reviewers conducted the potential risks of bias assessment using the Cochrane Collaboration tool. The risk of bias of a trial was assessed using seven items as follows: random sequence generation and allocation concealment (selection bias), blinding of participants and personnel (performance bias), blinding of outcome assessment (detection bias), incomplete outcome data (attrition bias), selective reporting (reporting bias), and other biases. Each item was classified as “low risk,” “high risk,” or “unclear risk.” Any disagreements were resolved by discussion with two reviewers. We pooled all results and performed subgroup analysis based on risk of bias to assess whether different risks of bias affected the estimate for data synthesis.

### 2.4. Data Analysis

Data are expressed as the mean ± standard deviation. Comparison of the differences among the variables of BCVA, CMT was done. Standard deviation was calculated according to the Cochrane Handbook formula (*R* = 0.5). We used the Review Manager software (version 5.3, Cochrane Community, UK) to analyze the data. Heterogeneity was assessed using the I^2^ test. The effects of BCVA and CMT were estimated using 95% confidence interval (CI) and weighted mean difference. If significant statistical heterogeneity existed in the pooled studies (*P* < 0.1, I^2^ > 50%), we adopted a random-effect model for meta-analysis; otherwise, a fixed-effect model was applied (*P* > 0.1, I^2^ ≤ 50%). Considering the follow-up time, a subgroup analysis was planned.

## 3. Results

### 3.1. Characteristics of Included Studies

**Figure 1:**
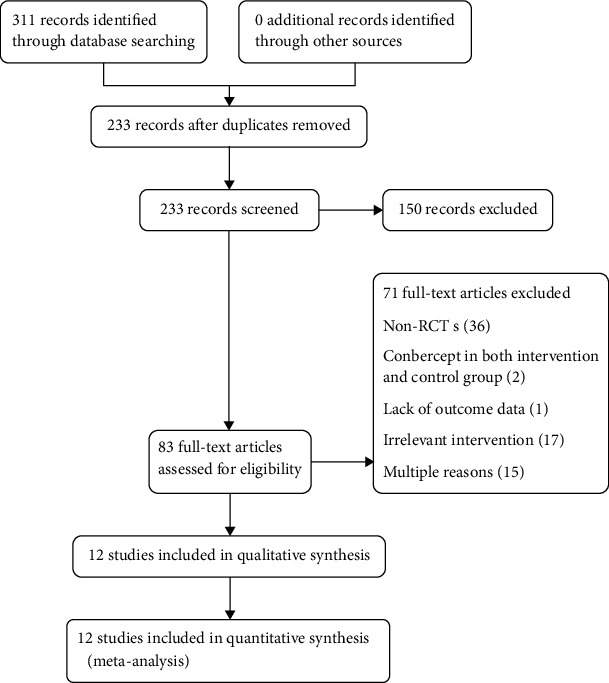
Flow diagram of included studies in this meta-analysis.

A total of 311 articles were identified by searching six electronic databases. The process of selecting research studies is shown in Figure 1. Finally, 12 articles [12–23] involving 2600 eyes with DME were enrolled in this meta-analysis. The main characteristics of the included studies are listed in Table 1. The follow-up time lasted from 1 week to 6 months after the initial treatment. The 12 articles enrolled in this meta-analysis reported the status of baseline and were comparable with each other. Three articles did not describe in detail the randomization methods. None of the articles explained whether allocation concealment was hidden. All the studies reported balanced baseline characteristics between the comparison groups. Three studies [13, 17, 23] presented BCVA in logMAR units, hence the outcomes could not be pooled in this meta-analysis. Statistical heterogeneity existed in several areas, such as follow-up selection and treatment regimen.

### 3.2. Risk of Bias in Included Articles

**Figure 2:**
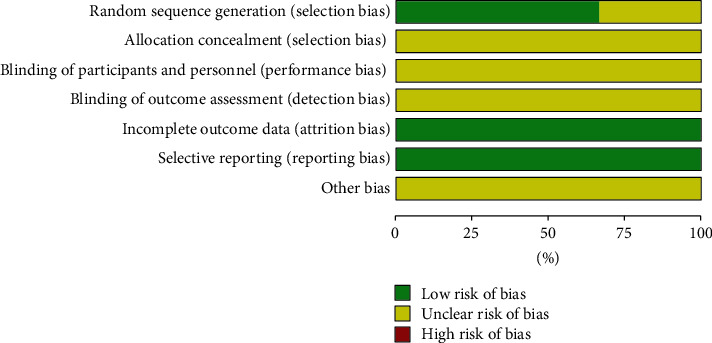
Risk of bias graph.

**Figure 3:**
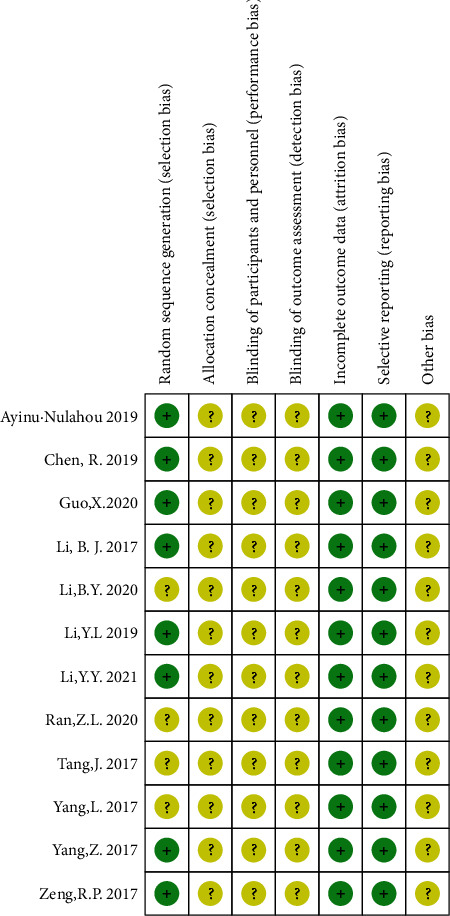
Risk of bias summary.

Generally, there was inadequate information available in the literature. All of the risk of bias assessment data are shown in Figures 2 and 3. The methodological quality of the trials was poor. The risk of bias of the articles was mostly “unclear risk.” None of the studies mentioned the blinding of participants and personnel, neither the blinding of outcome assessment.

### 3.3. Outcomes

#### 3.3.1. CMT

**Figure 4:**
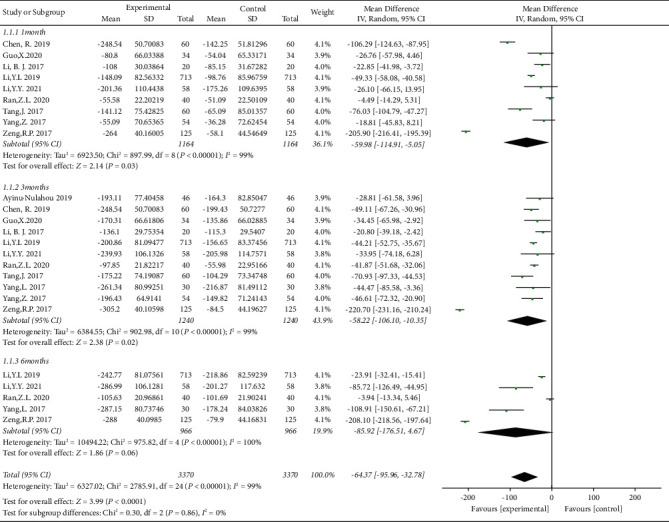
Forest plot showing the subgroup analyses on CMT stratified by follow-up selection.

There were 9 [12, 13, 15, 17, 18, 20–23], 11 [12–13, 15–23] and 5 [12, 15, 19, 21, 23] studies that reported the change in CMT from baseline and on follow-up after one, three, and six months. Subgroup analysis and stratification were performed according to the follow-up selection. It was found that the heterogeneity was significant; hence, the random-effects model was used to continue the analysis (Figure 4). There were significant differences among the pooled results of the three subgroups (*P* < 0.01). The pooled results revealed that IVC combined macular grid/focal photocoagulation significantly reduced CMT compared with macular grid/focal photocoagulation alone at 1 month (MD = −59.98; 95% CI: −114.91 to −5.05, *P* < 0.01) and 3 months (MD = −58.22, 95% CI: −106.10 to −10.35, *P* < 0.01). At 6 months, the difference was not significant (MD = −85.92, 95% CI: −176.51 to 4.67, *P*=0.06).

### 3.4. BCVA

**Figure 5:**
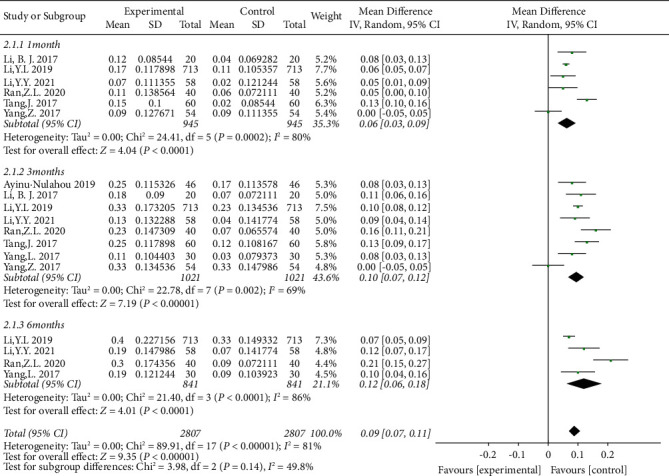
Forest plot showing the subgroup analyses on BCVA stratified by follow-up selection.

Six studies reported the mean changes in BCVA from baseline to 1 month after initial treatment [12, 15, 18, 20–22]. The outcomes at 3 months after initial treatment were reported in eight studies [12, 15, 16, 18–22], while the outcomes on the sixth month were reported in four studies [12, 15, 19, 21]. It was found that the heterogeneity was significant, so the random-effects model was selected for further analysis. Pooling the results revealed that the differences were significant among the three subgroups (*P* < 0.01) (Figure 5). Compared to the group of macular grid/focal photocoagulation alone, patients in the combination therapy group had significantly improved the BCVA in both the first month (MD = 0.06; 95% CI: 0.03 to 0.09, *P* < 0.01), third month (MD = 0.10; 95% CI: 0.07 to 0.12, *P* < 0.01), and sixth month (MD = 0.12; 95% CI: 0.06 to 0.18, *P* < 0.01) after treatment.

### 3.5. Effectiveness Rate

**Figure 6:**
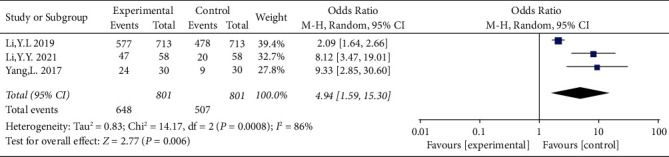
Forest plot showing the analyses on the effective rate in the two groups.

Three studies [15, 19, 21] (*n* = 1602) reported the effectiveness rate. Treatment effectiveness was defined as a VA improvement of ≥2 rows after treatment. There was significant heterogeneity among the studies (I^2^ = 86%, *P* < 0.01). A random-effects model should be selected to calculate the data. As shown in Figure 6, the effectiveness rate in the treatment group was better than that in the control group (OR = 4.94, 95% CI: 1.59 to 15.30, *P* < 0.01).

### 3.6. Complications

**Figure 7:**
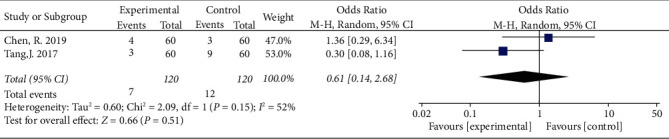
Forest plot showing the analyses on the incidence of complications in the two groups.

Three studies [17, 18, 22] (*n* = 240) reported complications. One study [18] did not present complications in terms of incidence; therefore, the data could not be pooled in this meta-analysis. Significant heterogeneity was present among the studies (I^2^ = 52%, *P*=0.15), and the random-effects model was applied (Figure 7). No significant differences in the incidence of complications were observed between the two groups (OR = 0.61, 95% CI: 0.14 to 2.68, *P*=0.51).

### 3.7. Publication Bias and Sensitivity Analysis

The sensitivity analysis showed that the study by Zeng et al. [21] had a great influence on the CMT of patients in the two groups after 3 months. After elimination, the differences were statistically significant between the two groups at 3 months (*P* < 0.01). The combination therapy tended to have a greater reduction (MD = −42.06, 95% CI: −48.94 to −35.17, *P* < 0.01). The study by Li et al. [16] had a great influence on the effectiveness rate in the two groups. Removal of this study did not change the results (MD = 8.51, 95% CI: 4.26 to 17.00, *P* < 0.01).

**Figure 8:**
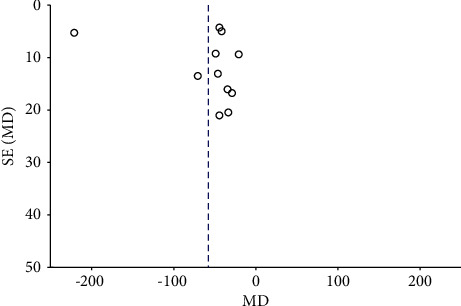
Inverted funnel plot of CMT at 3 months.

**Figure 9:**
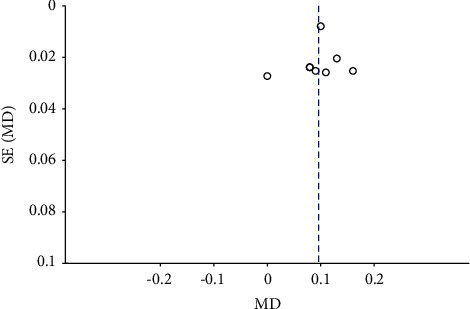
Inverted funnel plot of BCVA at 3 months.

The inverted funnel plot showed the difference in scatter symmetry on both sides of the line, suggesting that there was publication bias in the included studies (Figures 8 and 9).

### 3.8. Potential Biases in the Review

There were some areas in which bias may exist in this meta-analysis. There may be unpublished literature that was not retrieved although we have conducted a comprehensive literature search, and these studies may have a certain impact on the results. We emailed the researchers for information related to the study; however, none of the researchers responded. There was no explanation regarding the blinding method in the included studies. In addition, the subgroup analyses were conducted post hoc. This can lead to bias in the research.

## 4. Discussion

In our analysis, we found that the group that received combination therapy had a statistically significant improvement in BCVA and reduction in CMT over the control group, and this difference persisted for 3 months. At the later visit (6 months), the experimental group showed a significant improvement in BCVA, while no significant improvement in CMT was observed. These results showed that there was no absolute correlation between anatomical change (CMT) and functional change (BCVA). VA is only an assessment of central visual impairment and does not truly reflect visual function in the macular area. Hou et al. [22] pointed out that CMT is weakly associated with BCVA in DME. The integrity of the ellipsoid zone was closely associated with BCVA in DME. In another study conducted by Browning [23], age, glycosylated hemoglobin, and severity of fluorescein leakage in the center and inner subfields were responsible for the change in VA in addition to CMT. In this study, there was a large heterogeneity among the results of the included studies, which was mainly related to the differences in the severity of the disease, types of DME among the patients, doses and frequency of injections, and the macular laser photocoagulation method used. Due to the limited information available in the original literature, subgroup analysis by injection time, injection dose, and the macular laser photocoagulation method was not possible. The results of the inverted funnel plot of BCVA at 3 months revealed a publication bias, which may be caused increased likelihood of papers with positive results to be published than those with negative results. However, this meta-analysis has some limitations. First, all included studies were from China, and all the studies were conducted in a single center. Second, as a domestic innovative drug, conbercept is used in a few countries and the data were inadequate for a dose-response meta-analysis. Third, DR is a chronic disease which requires a longer follow-up time to observe the changes of the disease and summarize the rule of medication. At the same time, the languages of literature in this meta-analysis were limited to Chinese and English, and other languages and grey literature were not retrieved. In the future, further well-designed studies with higher quality are needed.

## 5. Conclusion

This meta-analysis confirms the superior efficacy of conbercept combined with macular grid/focal photocoagulation over macular grid/focal photocoagulation in patients with DME. Furthermore, patients in the combination therapy group had significantly reduced the area of CMT after treatment compared to the control group. In contrast, the patients in the combination therapy group showed better effectiveness. Therefore, combination therapy could be a potentially favorable treatment for DME.

## Figures and Tables

**Table 1:** Study characteristics of included studies.

Authors	Year	Study design	Intervention	Sampling	Age (year)	Jadad scale	Follow-up
Zeng, R.P.	2017	RCT	A	125	65.1 ± 7.1	4	1 mo, 3 mo, 6 mo
			B	125	64.8 ± 6.8		

Guo, X.	2020	RCT	IVC (3 injections, no mention of dose) + B	34	61.8 ± 2.4	4	1 wk, 1 mo, 3 mo
			B	34	62.7 ± 2.3		

Li, B.J.	2017	RCT	IVC (2 injections of 0.05 mL) + B	20	59.07 ± 5.12	4	45 d, 3 mo
			B	20	58.47 ± 4.29		

Li, B.Y.	2020	RCT	A	60	45.7 ± 7.3	3	15 d
			B	60	46.1 ± 6.6		

Li, Y,Y.	2021	RCT	A	58	64.39 ± 5.62	4	1 mo, 3 mo, 6 mo
			B	58	63.19 ± 6.12		

Li, Y.L.	2019	RCT	IVC (3 injections of 0.05 mL) + B	713	49.6 ± 12.8	4	1 wk, 1 mo, 3 mo, 6 mo
			B	713	48.3 ± 11.7		

Nulahou	2019	RCT	A	46	63.45 ± 6.63	4	3 mo
			B	46	62.10 ± 5.39		

Ran, Z.L.	2020	RCT	Single IVC (no mention of dose) + B	40	60.95 ± 9.24	3	1 mo, 3 mo, 6 mo
			B	40	60.31 ± 8.37		

Tang, J.	2017	RCT	A	60	51.56 ± 1.48	3	1 mo, 2 mo, 3 mo
			B	60	52.64 ± 1.58		

Yang, L.	2017	RCT	single IVC (no mention of dose) + B	30	62.61 ± 6.59	4	3 mo, 6 mo
			B	30	63.14 ± 6.79		

Yang, Z.	2017	RCT	IVC (3 injections of 0.05 mL) + B	54	51.68 ± 10.02	4	1 mo, 2 mo, 3 mo
			B	54	51.60 ± 11.48		

Chen, R.	2019	RCT	IVC (3 injections of 0.05 mL) + B	60	56.72 ± 6.88	4	1 mo, 3 mo
			B	60	56.27 ± 6.82		

A: single IVC (0.05～0.1 mL) + B; B: macular focal/grid photocoagulation.

## Data Availability

The data used to support the findings of this study are available from the corresponding author upon request.
